# Model Free Approach for Non-Isothermal Decomposition of Un-Irradiated and γ-Irradiated Silver Acetate: New Route for Synthesis of Ag_2_O Nanoparticles

**DOI:** 10.3390/ijms11093600

**Published:** 2010-09-27

**Authors:** Mohmmed Rafiq H. Siddiqui, Saad Alshehri, Ismail Kh. Warad, Naser M. Abd El-Salam, Refaat M. Mahfouz

**Affiliations:** 1 Department of Chemistry, College of Science, King Saud University, P. O. Box 2455, Riyadh-11451, Saudi Arabia; E-Mails: rafiqs@ksu.edu.sa (M.R.H.S.); alshehri@ksu.edu.sa (S.A.); 2 Department of Natural Sciences, Riyadh Community College, King Saud University, P. O. Box 28095, Riyadh-11437, Saudi Arabia; E-Mail: nelsalam@yahoo.com

**Keywords:** non-isothermal decomposition, γ-irradiation, silver oxide, nanoparticles

## Abstract

Kinetic studies for the non-isothermal decomposition of unirradiated and γ-irradiated silver acetate with 10^3^ kGy total γ-ray doses were carried out in air. The results showed that the decomposition proceeds in one major step in the temperature range of (180–270 °C) with the formation of Ag_2_O as solid residue. The non-isothermal data for un-irradiated and γ-irradiated silver acetate were analyzed using Flynn-Wall-Ozawa (FWO) and nonlinear Vyazovkin (VYZ) iso-conversional methods. These free models on the investigated data showed a systematic dependence of Ea on α indicating a simple decomposition process. No significant changes in the thermal decomposition behavior of silver acetate were recorded as a result of γ-irradiation. Calcinations of γ-irradiated silver acetate (CH_3_COOAg) at 200 °C for 2 hours only led to the formation of pure Ag_2_O mono-dispersed nanoparticles. X-ray diffraction, FTIR and SEM techniques were employed for characterization of the synthesized nanoparticles.

## 1. Introduction

Thermal treatment of inorganic substances has great synthetic potential as it may turn simple compounds into advanced materials, such as ceramics, catalysts, and glass and could reproducibly lead in most cases to metal or metal oxide nanoparticles displaying a very narrow size distribution [1]. Many recent studies on the thermal decomposition of inorganic solids have included measurements on samples that were exposed to radiation prior to heating with the air to investigate the effect of ionizing radiation on the kinetics and thermal decomposition behavior of inorganic compounds [2]. Silver acetate is a suitable metallic organic compound for preparation of silver nanoparticles for medical applications such as diagnostic biomedical optical imaging, and biological implants like heart valves [3].

In the present investigation, thermal decomposition behavior of un-irradiated and γ-irradiated silver acetate was investigated in air with the aim to (a) examine the kinetics of non-isothermal, decomposition of both unirradiated and γ-irradiated silver acetate using a model free iso-conversional approach and compare the results with those obtained in our previously reported investigation using model fitting method [4] (b) to clarify the effects of, γ-irradiation on the thermal decomposition behavior, and (c) finally to follow the chemical composition of the solid residue obtained at different temperatures while attempting to prepare Ag_2_O at nanoscale by thermal decomposition of silver acetate in the air.

## 2. Results and Discussion

**Figure 1:**
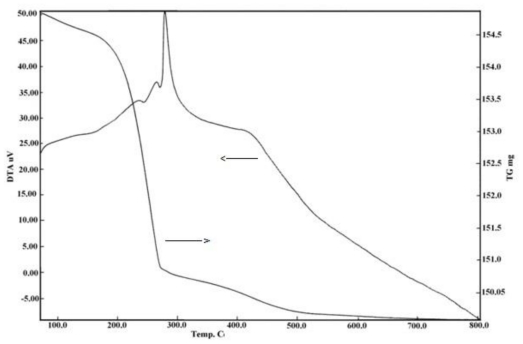
TG and DTA of silver acetate.

The TG curve showed that decomposition of silver acetate (CH_3_COOAg) proceeds, in one major decomposition step in the temperature range of 200–280 °C, the formation of Ag(0) as solid residue. Non-isothermal decomposition of silver acetate was previously studied in detail in an inert atmosphere [5]. This decomposition step was associated with two minor and one major sharp endothermic peaks as shown in DTA curve in Figure 1. This indicates that the main decomposition step probably contains three overlapping decomposition steps. A weight gain was detected in the temperature of 300–400 °C attributed to oxidation of Ag(0) and formation of silver oxide Ag_2_O as solid product. This weight gain was characterized by a sharp exothermic peak centered at ≈350 °C. Minor decomposition was recorded after 400 °C and up to 500 °C associated with broad exothermic peak and could be ascribed to formation of non-stoichiometric silver oxide.

No significant changes were detected with the thermal decomposition behavior of silver acetate as a result of γ-irradiation up to 10^3^ kGy total γ-ray dose absorbed by the sample, except that the onset temperature was found at a relatively lower temperature by about 20 °C for γ-irradiated sample.

**Figure 2:**
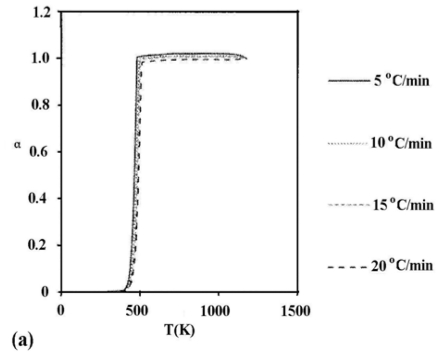

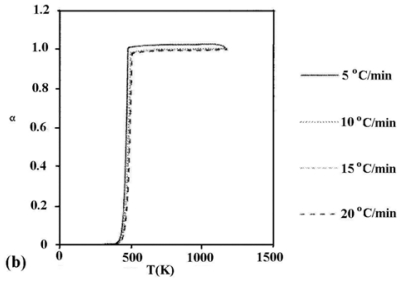
Thermogravimetric data showing the extent of (**a**) un-irradiated and (**b**) γ-irradiated sliver acetate conversion during non-isothermal decomposition.

**Figure 3:**
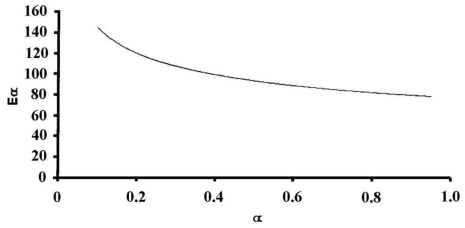
Activation energy Ea Vs α curves for un-irradiated and γ-irradiated sliver acetate.

The dynamic data for both un-irradiated and γ-irradiated silver acetate with 10^3^ kGy γ-ray dose are shown in Figure 2. The application of both linear (EWZ) and non-linear (VYZ) model free iso-conversional methods, to the non-isothermal decomposition data for both un-irradiated and γ-irradiated silver acetate with 10^2^ kGy total γ-ray dose, permits a determination of E_a_ as a function of α, and the results are shown in Figure 3, where the values of activation energies for both unirradiated and gamma irradiated samples are almost identical and, therefore, we represent the variation in one single curve. The results of the application of these two model free approaches on the investigated data in general showed a systematic dependence of E_a_ on the extent of conversion of silver acetate α, indicating a simple decomposition process with almost the same estimated values of E_a_ using two free approach models on the linear (FWO) and non-linear (VYZ) methods: E_a_ is ≈140 kJ mol^−1^ and 120 kJ mol^−1^ at the beginning of decomposition, then reaches a minimum of 80 kJ mol^−1^ at the end of the decomposition for both un-irradiated and γ-irradiated samples. The relative lower E_a_ value obtained at the start of decomposition for γ-irradiated sample could be attributed to the formation of additional decomposition centers created by trapped electrons and holes in the host lattice.

### 2.1. IR, XRD, SEM and TEM Measurements

IR spectra of both un-irradiated and γ-irradiated silver acetate display the characteristic bands assigned to various vibration modes of acetate functional groups. Neither disappearance nor appearance of new bands was recorded in the IR spectrum of γ-irradiated silver acetate with 10^3^ kGy total γ-ray dose, but a decrease in the intensity of most characteristic bands was recorded as a result of γ-irradiation. The band assigned to ν_Ag-O_ is around 550 cm^−1^ and was more affected by irradiation than any other band in the spectrum. The decrease in the intensity of this band could be attributed to bond scission and/or degradation caused by γ-irradiation.

**Figure 4:**
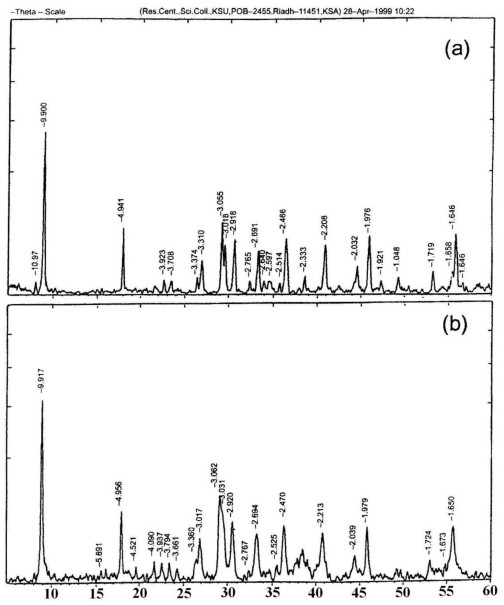
XRD for sliver acetate (**a**) un-irradiated and (**b**) γ-irradiated (10^3^ kGy).

XRD patterns of un-irradiated and γ-irradiated silver acetate are shown in Figure 4a,b, respectively, both showed the same characteristic features of monoclinic system without any significant changes occurring as a result of γ-irradiation with 10^3^ kGy total γ-ray dose.

### 2.2. Role of Irradiation

Upon irradiation with ^60^Co γ-ray, the Compton effect has the largest cross-section, except for materials of very high atomic number and, moreover, the number of atoms displaced has its maximum effect in the very light elements and diminishes to zero around atomic number 125 [6]. γ-Irradiation generates additional sites of potential nucleation. These may be crystal defects or reactive radicals that are not necessarily identical with intrinsic nucleation sites but are of comparable reactivity and, or most probably, evolve by a similar sequence of steps into nuclei growth. More extensive irradiation shifts advance the onset of reaction; this is envisaged as being due to the involvement of a small amount of decomposition products, which promote the transformation of all precursor-specific sites into active growth nuclei. The kinetic of growth of all nuclei are identical. The observed increase in reaction rate for γ-irradiated samples is ascribed to a direct relationship between the extent of salt, γ-irradiation and number of nuclei developed on subsequent decomposition [7].

**Figure 5:**
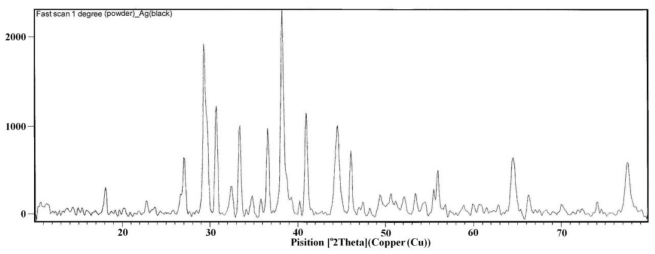
XRD pattern for synthesized silver oxide nanoparticles.

**Figure 6:**
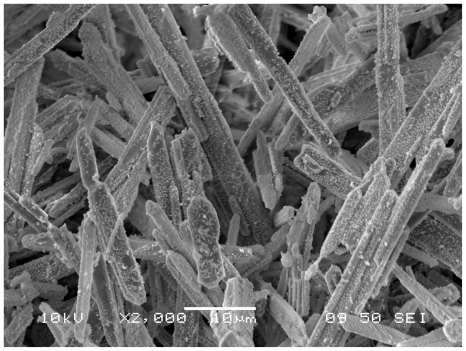
SEM image of sliver oxide nanoparticles.

**Figure 7:**
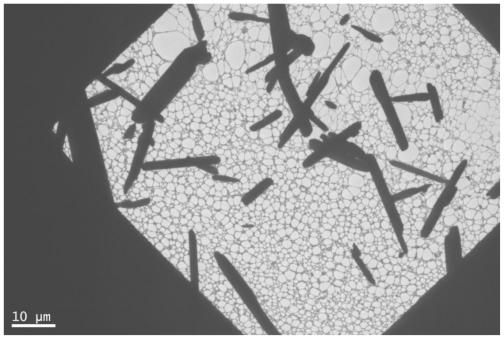
TEM image of sliver oxide nanoparticles.

Figure 5 shows the representative XRD pattern of as-synthesized silver oxide nanoparticles. All diffraction lines are indexed to monoclinic Ag_2_O. Figure 6 shows SEM image of the sample. It can be seen that Ag_2_O is present as nanorods over which tiny and agglomerated particles are formed. The morphology of prepared nanoparticles was studied by TEM. Figure 7 shows the typical image for Ag_2_O nanoparticles. These nanoparticles have an average diameter of 2–3 nm. In the TEM images, the shape of these nanoparticles is rod- like, the same as observed in SEM images without any evidence of aggregation.

IR spectrum of as-prepared Ag_2_O nanoparticles display the characteristic band attributed to lattice vibration of Ag_2_O at 550 cm^−1^.

## 3. Experimental Section

Silver acetate with 99.999% (wt.%) purity was obtained commercially from (BDH, UK). The sample used for investigation was dried at 200 °C to ensure complete dehydration.

The decomposition of silver acetate in air yields Ag_2_O and volatile products, and, within the experimental error, eventually goes to completion. The weights of investigated samples were in the range of 100 mg. The decomposition followed in air using dynamic thermogravimetric techniques in the temperature range (25–800 °C).

Under dynamic non-isothermal conditions four linear heating rates (5, 10, 15, 20 °C/min) were applied. For irradiation experiments, samples were encapsulated under vacuum in glass vials and exposed to successively increasing doses of radiation at constant intensity using a cobalt-60 γ-ray cell 220 (Nordion, INT-INC, Ontario, Canada) at a dose rate of 10^4^ Gy/h. The source was calibrated against a Fricke ferrous sulfate dosimeter and the dose rate in the irradiated samples was calculated by applying appropriate corrections on the basis of both the photon mass attenuation and energy absorption coefficient for the sample and dosimeter [8]. The fraction decomposed or the extent of conversion α was calculated as:

$$\alpha =\frac{M_{0}-M}{M-M_{f}}$$

where M_0_, M, M_f_ are the initial, actual and final sample mass, respectively.

### 3.1. Synthesis of Ag_2_O Nanoparticles

A sample of γ-irradiated silver acetate with a 10^3^ kGy total γ-ray dose was heated for 2 hours in a furnace at 200 °C where a black residue was obtained. The two separate solid residues were collected and subjected to analysis using solid state characterization techniques including XRD, FTIR, SEM and TEM measurements. A parallel experiment was performed starting with un-irradiated silver acetate, where the solid black residue was observed after heating for 24 hours at 300 °C.

The infrared spectra were recorded in KBr pellets using a Perkin-Elmer 1000FT-IR spectrophotometer.

XRD measurements were carried out on a Siemens D5000 X-ray diffractometer using a nickel filter (Cu Kα at λ = 1.5418 Å).

SEM was performed using JSM 6360 ASEM instrument. TEM was performed using TEM-200 CX instrument (Japan).

Further experiments concerning preparation of silver oxide nanoparticles using sol-gel methods starting from un-irradiated and γ-irradiated silver acetate and silver acetyl acetonate are now in progress at our laboratory at King Saud University.

**Theory**: a single step process for solid state decomposition has the following kinetic equations:

(1) $$\frac{d\alpha }{dt}=k(t)f(\alpha )$$

where α is the extent of conversion, k(t) is a temperature dependent reaction rate constant and f(α) the kinetics dependent model function. The Arrhenius equation expresses the explicit temperature dependency of the rate constant as:

(2) $$\frac{d\alpha }{dt}=A\text{exp}\left(\frac{-E_{a}}{RT}\right)f(\alpha )$$

The A, E_a_ and f(α) are called the kinetic triplet that can characterize a unique decomposition reaction. Under non-isothermal conditions in which a sample is heated at a constant rate, the explicit time dependence in Equation (1) is eliminated through the trivial transformation:

(3) $$\frac{d\alpha }{dt}=\frac{A}{\beta }\left(\frac{-E_{a}}{RT}\right)f(\alpha )$$

where β = dT/dt is the heating rate.

Upon integration, Equation (1) gives:

(4) $$\begin{array}{l} g(\alpha )=\int_{0}^{a}\frac{d\alpha }{f(\alpha )}=\frac{A}{\beta }\int_{0}^{T}\text{exp}\left(\frac{-E_{a}}{RT}\right)f(\alpha )\\ \approx \frac{A}{\beta }\int_{0}^{T}\text{exp}\left(\frac{-E_{a}}{RT}\right)dT=\frac{{AE}_{a}}{R\beta }\int_{0}^{x_{\alpha }}\text{exp}\left(\frac{-x}{x^{2}}\right)dx\\ =\frac{{AE}_{a}}{R\beta }p(x)\equiv \frac{A}{\beta }I(E_{a},T) \end{array}$$

where P(x) is the exponential integral for x = Ea/RT. The temperature integral I (E_α_, T) has no analytical solution but many approximations.

The linear iso-conversional integral method, suggested independently by Flynn and Wall and Ozawa, uses Dolyle’s approximation of P(x) [9]. The method is based on equation:

(5) $$\text{ln}\beta =\text{ln}\left(\frac{{AE}_{0}}{R_{g}(\alpha )}\right)-5.3305-1.052\left(\frac{E_{a}}{RT}\right)$$

Thus for α = constant. The plot lnβ *versus* (1/T), obtained from thermograms recorded at several heating rates, should be a straight line, the slope of which can be used to evaluate the apparent activation energy E_a_.

A nonlinear iso-conversional method has been developed by Vyazovekin that avoids inaccuracies associated with analytical approximation of the temperature integral [10], for a set of n experiments carried out at different heating rates, the activation energy can be determined at any particular value of α by finding the value of E_a_ for which the objective function Ω is minimized, where:

(6) $$\begin{array}{l} \Omega =\sum_{i=1}^{n}\sum_{j\neq i}^{n}\frac{I(E_{a,\alpha },T_{a,i})\beta_{j}}{I(E_{a,\alpha },T_{a,j})\beta_{i}}\\ I(E_{a,\alpha },T_{a,i})=\int_{0}^{t_{\alpha },i}\text{exp}\left(-\frac{E_{a,\alpha }}{RT}\right)dT \end{array}$$

We have developed a mathematical program to evaluate the temperature integral and the results have been compared with Cai *et al*. [11] approximation where:

(7) $$\int_{0}^{T}\text{exp}\left(-\frac{E_{a,\alpha }}{RT_{\alpha ,i}}\right)dT=\frac{{RT}_{a,\alpha ,i}^{2}}{E_{a,\alpha }}\left[\frac{\frac{E_{a,\alpha }}{RT_{\alpha ,i}}+0.66691}{\frac{E_{a,\alpha }}{RT_{\alpha ,i}}+2.64943}\right]\text{exp}\left(\frac{-E_{a,\alpha }}{RT_{\alpha ,i}}\right)$$

## 4. Conclusions

No significant changes were observed in the thermal decomposition behavior between unirradiated and γ-irradiated samples. Calcinations of γ-irradiated silver acetate (CH_3_COOAg) at 200 °C for 2 hours only led to the formation of Ag_2_O mono-dispersed nanoparticles. Attempts to prepare silver oxide nanoparticles using unirradiated samples under the same conditions were unsuccessful. The SEM and TEM show the formation of nanorods. The TEM shows the diameter of these rods to be between 2–3 nm. To our knowledge this is the first report to prepare Ag_2_O nanoparticles by solid state thermal decomposition of γ-irradiated silver acetate.
